# Quantitative Insights Into β-Lactamase Inhibitor’s Contribution in the Treatment of Carbapenemase-Producing Organisms With β-Lactams

**DOI:** 10.3389/fmicb.2021.756410

**Published:** 2021-11-18

**Authors:** Yanfang Feng, Arend L. de Vos, Shakir Khan, Mary St. John, Tayyaba Hasan

**Affiliations:** ^1^Wellman Center for Photomedicine, Massachusetts General Hospital, Harvard Medical School, Boston, MA, United States; ^2^Swammerdam Institute for Life Sciences, University of Amsterdam, Amsterdam, Netherlands; ^3^Department of Physics, University of Massachusetts, Boston, MA, United States; ^4^School of Arts and Sciences, Tufts University, Medford, MA, United States; ^5^Health Sciences and Technology (Harvard-MIT), Cambridge, MA, United States

**Keywords:** carbapenemase, β-lactamase inhibitor, antimicrobial stewardship, β-lactam antibiotics, carbapenem resistance

## Abstract

**Objectives:** Carbapenemase-producing organisms (CPOs) are associated with high mortality rates. The recent development of β-lactamase inhibitors (BLIs) has made it possible to control CPO infections safely and effectively with β-lactams (BLs). This study aims to explicate the quantitative relationship between BLI’s β-lactamase inhibition and CPO’s BL susceptibility restoration, thereby providing the infectious disease society practical scientific grounds for regulating the use of BL/BLI in CPO infection treatment.

**Methods:** A diverse collection of human CPO infection isolates was challenged by three structurally representative BLIs available in the clinic. The resultant β-lactamase inhibition, BL susceptibility restoration, and their correlation were followed quantitatively for each isolate by coupling FIBA (fluorescence identification of β-lactamase activity) and BL antibiotic susceptibility testing.

**Results:** The β-lactamase inhibition and BL susceptibility restoration are positively correlated among CPOs under the treatment of BLIs. Both of them are dependent on the target CPO’s carbapenemase molecular identity. Of note, without sufficient β-lactamase inhibition, CPO’s BL susceptibility restoration is universally low across all tested carbapenemase molecular groups. However, a high degree of β-lactamase inhibition would not necessarily lead to a substantial BL susceptibility restoration in CPO probably due to the existence of non-β-lactamase BL resistance mechanisms.

**Conclusion:** BL/BLI choice and dosing should be guided by quantitative tools that can evaluate the inhibition across the entire β-lactamase background of the CPO upon the BLI administion. Furthermore, rapid molecular diagnostics for BL/BLI resistances, especially those sensitive to β-lactamase independent BL resistance mechanisms, should be exploited to prevent ineffective BL/BLI treatment.

## Introduction

Carbapenemase-producing organisms (CPOs) are multidrug-resistant pathogens associated with high mortality rates (13.3–67%; Tamma et al., 2017). The recent development of β-lactamase inhibitors (BLIs) that could inhibit carbapenemases, the most potent type of β-lactamases, has made it possible to control CPO infections safely and effectively with β-lactam (BL) antibiotics (Cui et al., 2019; El Hafi et al., 2019; Sheu et al., 2019; Pogue et al., 2020). Unfortunately, recent clinical data have emerged demonstrating that treatment failures and subsequent bacterial resistance development may occur in CPO treatments with BLs and novel BLIs, necessitating further development of guidelines on rational use of BLs and BLIs for CPO infections (Shields et al., 2016; Cui et al., 2020).

Clinically, BLIs are available together with β-lactam antibiotics (BLs) at fixed dosages, forming the so-called BL/BLI agents. The rational choice of BL/BLI for CPO infections could appear straightforward according to the knowledge of each BLI’s carbapenemase inhibitory spectrum and carbapenemase type(s) of the CPO. However, whether the desired anti-CPO effect of the chosen BL/BLI agent could be achieved across diverse pathogens by a fixed BLI dosage is still of great concern for the infectious disease society (Spellberg and Bonomo, 2016) due to the large variety of CPO carbapenemase molecular structures and expression statuses. Moreover, many CPO co-produce other β-lactamases, including extended-spectrum β-lactamase (ESBL) and AmpC β-lactamase, both of which can reduce BLI activity (Ferreira et al., 2020; Shields and Doi, 2020). Additionally, the further complicating effects of other BL resistance mechanisms unrelated to β-lactamase (e.g., efflux pump overproduction, drug target alterations and porin mutations) and their influence on CPO response to BL/BLI agents remain unclear (Karumathil et al., 2018; Nicolas-Chanoine et al., 2018; Nordmann and Poirel, 2019; Black et al., 2020).

Our technology, fluorescence identification of β-lactamase activity (FIBA), has now enabled the quantification of β-lactamase activity in CPO regardless of their β-lactamase backgrounds (Sallum et al., 2010; Erdem et al., 2014; Khan et al., 2014; Feng et al., 2020, 2021). It uses β-lactamase enzyme-activated fluorophore (β-LEAF), which turns from dark to fluorescent when cleaved by β-lactamases, such as penicillinases, ESBL, AmpC β-lactamases, and carbapenemases. Therefore, the fluorescence increase rate (R) of β-LEAF is a direct measure of the activity of bacterial β-lactamases, and R is decreased as the β-lactamase activity is inhibited by BLIs. By coupling FIBA with BL susceptibility detection, this study aims to explicate the quantitative relationship between β-lactamase inhibition and BL susceptibility restoration in CPO under BLI treatment, thereby providing a detailed insight into the contribution of BLIs in CPO treatment with BLs. To this end, a diverse collection of human CPO infection isolates was challenged by three structurally representative BLIs available in the clinic (clavulanate, vaborbactam, and avibactam) to generate broadly applicable conclusions. The resultant β-lactamase inhibition, BL susceptibility restoration, and their quantitative correlation were investigated for different carbapenemase molecular groups to assist the future development of the current carbapenemase molecular type-derived BL/BLI administration guidelines for CPO infections.

## Materials and Methods

### Bacterial Isolates, BL and BLIs

Bacterial isolates of human CPO infection were acquired from the CDC and FDA Antibiotic Resistance Isolate Bank. The BL resistance mechanisms, including the β-lactamase production, were identified by analyzing the whole genome sequence of the isolates with the Resistance Gene Identifier of the Comprehensive Antibiotic Resistance Database (McArthur et al., 2013; Alcock et al., 2020). The information of the genome sequences (i.e., sequence accession numbers) of the tested isolates was provided by the CDC and FDA Antibiotic Resistance Isolate Bank, and is available on the official website of this isolate bank. Three BLIs (clavulanate, vaborbactam, avibactam) and their clinically combined partner BLs (amoxicillin for clavulanate; meropenem for vaborbactam; ceftazidime for avibactam) were purchased from Sigma-Aldrich. To facilitate comparison, all BLIs were tested at the same concentration attainable in patient plasma (50μm) (Carlier et al., 2013; Nicolau et al., 2015; Lee et al., 2019).

### Quantification of β-Lactamase Inhibition

BLI’s β-lactamase inhibition was quantified by β-lactamase inhibition index (BI), which is defined as the ratio of β-lactamase activity with and without BLI. The β-lactamase activity was measured by FIBA as previously described (Feng et al., 2021). Briefly, bacterial culture was exposed to β-lactamase enzyme-activated fluorophore (β-LEAF), and the fluorescence increase rate (R), which is the direct measure of the β-lactamase activity, was then acquired by monitoring the β-LEAF fluorescence every 10s for 10min with an excitation wavelength of 450nm and an emission wavelength of 510nm at 37°C. Thus, BI could be acquired by the equation below:


$$\mathrm{BI}=R^{\mathrm{without}\;\mathrm{BLI}}/R^{\mathrm{with}\;\mathrm{BLI}}$$

### BL Susceptibility Restoration

The BL susceptibility, with and without the three tested BLIs, was determined by measuring the minimal inhibitory concentration (MIC) with the broth microdilution method following the Clinical and Laboratory Standards Institute guidelines. The MIC reduction of BL due to the addition of BLI was defined as the BL susceptibility restoration in response to the tested BLI. An isolate with BL MIC reduced 4-fold was considered sensitized toward the tested BL by the inclusion of the tested BLI.

### Statistical Analysis

Data analysis was performed in R (v3.6.3). BI and BL susceptibility restoration was compared among BLIs using Kruskal-Wallis followed by *post hoc* pairwise Dunn testing. The quantitative correlation of BI with the ratio of sensitized isolates for each BLI was analyzed by the polynomial regression model. All statistical analyses were considered significant at a value of *p*<0.05.

## Results

This study tested CPO infection isolates from 15 different species, including *Pseudomonas aeruginosa*, *Acinetobacter baumannii*, and 13 *Enterobacteriaceae* species, as shown in Tables 1–3. These isolates produce a range of carbapenemase molecular classes commonly found in clinic, including Class A carbapenemases (*n*=43, Table 1), Class B carbapenemases (*n*=45, Table 2), and Class D carbapenemases (*n*=73, Table 3). Besides carbapenemases, many of the tested isolates co-produce non-carbapenemase β-lactamases, such as ESBL (59%) and AmpC β-lactamase (84%), reflecting CPO’s “complex β-lactamase background” encountered in clinic. In addition to β-lactamase, other β-lactam resistance mechanisms, such as overexpression of efflux pumps, inactivation of drug target, and porin mutations, are also found among most of the tested isolates (Tables 1–3), illustrating the multifactorial nature of BL resistance in CPO.

**Table 1:** Class A carbapenemase-producing isolates included in this study.

Carba subtype	Species	BL susceptibility (With/without BLI)			Other BL resistance Mechanisms		
		AMX/CA	MPN/VB	CFZ/AV	Efflux pumps	Reduced permeability	Target alteration
KPC	*C. freundii*	>1024/>1024	512/≤0.5	>1024/≤0.5	Y	Y	Y
	*E. cloacae*	>1024/1024	32/32	>1024/4	Y	Y	Y
		>1024/>1024	256/≤0.5	>1024/≤0.5	Y	Y	Y
		>1024/>1024	64/≤0.5	1024/≤0.5	Y	Y	Y
		>1024/>1024	>1024/1	>1024/256	Y	Y	Y
		>1024/>1024	64/≤0.5	1024/≤0.5	Y	Y	Y
		>1024/>1024	64/≤0.5	512/1	–	–	–
		>1024/>1024	128/≤0.5	>1024/2	–	–	–
		>1024/>1024	64/≤0.5	>1024/2	–	–	–
		>1024/>1024	256/1	>1024/2	–	–	–
	*E. coli*	>1024/1024	16/≤0.5	≤0.5/≤0.5	Y	Y	Y
		>1024/>1024	128/64	>1024/4	Y	Y	Y
		>1024/>1024	16/≤0.5	>1024/≤0.5	Y	Y	Y
		>1024/>1024	128/≤0.5	256/≤0.5	–	–	–
	*K. oxytoca*	>1024/1024	32/≤0.5	>1024/≤0.5	Y	Y	Y
	*K. ozaenae*	>1024/>1024	256/≤0.5	128/≤0.5	Y	Y	Y
	*K. pneumoniae*	>1024/>1024	1024/16	>1024/1024	Y	Y	Y
		>1024/>1024	1024/≤0.5	256/≤0.5	Y	Y	Y
		>1024/>1024	1024/16	>1024/8	Y	Y	Y
		>1024/>1024	>1024/128	>1024/8	Y	Y	Y
		>1024/>1024	1024/16	>1024/8	Y	Y	Y
		>1024/>1024	1024/2	>1024/8	Y	Y	Y
		>1024/>1024	>1024/64	>1024/1024	Y	Y	Y
		>1024/>1024	>1024/32	>1024/>1024	Y	Y	Y
		>1024/>1024	512/8	512/2	Y	Y	Y
		>1024/4	32/8	1024/≤0.5	Y	Y	Y
		>1024/>1024	128/≤0.5	128/≤0.5	–	–	–
		>1024/>1024	4/≤0.5	512/≤0.5	–	–	–
		256/256	128/≤0.5	16/≤0.5	–	–	–
		>1024/>1024	256/4	128/2	–	–	–
		>1024/>1024	1024/8	>1024/2	–	–	–
		>1024/>1024	256/4	>1024/1	–	–	–
		>1024/>1024	1024/32	>1024/1	–	–	–
		>1024/>1024	512/16	1024/2	–	–	–
		>1024/>1024	128/≤0.5	>1024/0.5	–	–	–
		>1024/256	512/4	256/1	–	–	–
		>1024/>1024	128/2	1024/1	–	–	–
		>1024/>1024	64/1	64/≤0.5	–	–	–
		>1024/>1024	64/≤0.5	256/≤0.5	–	–	–
	*M. morganii*	>1024/>1024	16/≤0.5	>1024/2	Y	N	Y
	*P. mirabilis*	>1024/>1024	256/128	>1024/1024	Y	N	Y
		512/64	4/4	128/128	Y	N	Y

Carba, carbapenemase; β-lactams (BLs) tested include CA (clavulanate), VB (vaborbactam), AV (avibactam); Partner β-lactamase inhibitors (BLIs) tested include CA (clavulanate), VB (vaborbactam), AV (avibactam); −, Not available.

**Table 2:** Class B carbapenemase-producing isolates included in this study.

Carba subtype	Species	BL susceptibility (With/without BLI)			Other BL resistance Mechanisms		
		AMX/CA	MPN/VB	CFZ/AV	Efflux pumps	Reduced permeability	Target alteration
IMP	*K. aerogenes*	>1024/≤0.5	32/32	1024/64	Y	Y	Y
	*K. pneumoniae*	>1024/>1024	128/128	>1024/>1024	Y	Y	Y
	*P. aeruginosa*	>1024/>1024	1024/1024	>1024/1024	Y	N	Y
		>1024/>1024	>1024/>1024	>1024/>1024	Y	N	Y
NDM	*A. baumannii*	>1024/>128	512/512	>1024/>1024	Y	N	N
	*C. freundii*	>1024/>1024	>1024/>1024	>1024/1024	Y	Y	Y
	*E. coli*	>1024/>1024	>1024/>1024	>1024/>1024	Y	Y	Y
		>1024/>1024	>1024/>1024	>1024/>1024	Y	Y	Y
		>1024/>1024	8/≤0.5	>1024/1024	Y	Y	Y
		>1024/>1024	512/512	>1024/≤0.5	Y	Y	Y
		>1024/>1024	256/256	>1024/>1024	Y	Y	Y
		>1024/>1024	1024/1024	>1024/>1024	Y	Y	Y
		>1024/>1024	1024/1024	>1024/>1024	Y	Y	Y
		>1024/>1024	>1024/>1024	>1024/>1024	Y	Y	Y
		>1024/≤0.5	1024/1024	>1024/≤0.5	Y	Y	Y
		>1024/≤0.5	>1024/>1024	>1024/≤0.5	Y	Y	Y
		>1024/>1024	512/512	>1024/>1024	Y	Y	Y
	*K. pneumoniae*	>1024/512	512/512	>1024/>1024	Y	Y	Y
		>1024/>1024	1024/1024	>1024/512	Y	Y	Y
		>1024/>1024	256/256	>1024/>1024	Y	Y	Y
		>1024/>1024	>1024/>1024	>1024/>1024	Y	Y	Y
		>1024/>1024	>1024/>1024	1024/1024	Y	Y	Y
		>1024/≤0.5	512/512	>1024/256	Y	Y	Y
		>1024/>1024	1024/1024	>1024/>1024	Y	Y	Y
		>1024/>1024	512/512	>1024/>1024	Y	Y	Y
		>1024/>1024	1024/1024	>1024/>1024	Y	Y	Y
		>1024/>1024	>1024/>1024	>1024/>1024	Y	Y	Y
		>1024/>1024	512/512	>1024/512	Y	Y	Y
		>1024/>1024	1024/1024	>1024/>1024	Y	Y	Y
	*M. morganii*	>1024/>1024	16/8	>1024/>1024	Y	N	Y
	*P. mirabilis*	>1024/>1024	512/512	>1024/>1024	Y	N	Y
	*P. rett*geri	>1024/>1024	>1024/>1024	>1024/>1024	Y	N	Y
	*S. senftenberg*	>1024/>1024	1024/1024	>1024/>1024	Y	Y	Y
SPM	*P. aeruginosa*	>1024/>1024	>1024/>1024	>1024/>1024	Y	N	Y
VIM	*E. cloacae*	>1024/>1024	>1024/>1024	>1024/>1024	Y	Y	Y
	*K. pneumoniae*	>1024/>1024	>1024/>1024	>1024/>1024	Y	Y	Y
		>1024/>1024	>1024/>1024	>1024/>1024	N	N	N
		>1024/>1024	>1024/>1024	128/128	Y	Y	Y
		>1024/>1024	>1024/>1024	>1024/>1024	Y	Y	Y
	*P. aeruginosa*	>1024/>1024	>1024/>1024	>1024/>1024	Y	N	Y
		>1024/>1024	512/512	256/128	Y	N	Y
		>1024/>1024	>1024/1024	>1024/>1024	Y	N	Y
		>1024/>1024	512/512	>1024/64	Y	N	Y
		>1024/>1024	256/256	64/16	Y	N	Y

Carba, carbapenemase; β-lactams (BLs) tested include CA (clavulanate), VB (vaborbactam), AV (avibactam); Partner β-lactamase inhibitors (BLIs) tested include CA (clavulanate), VB (vaborbactam), AV (avibactam).

**Table 3:** Class D Carbapenemase-producing isolates included in this study.

Carba Subtype	Species	BL susceptibility (With/without BLI)			Other BL resistance Mechanisms		
		AMX/CA	MPN/VB	CFZ/AV	Efflux pumps	Reduced permeability	Target alteration
OXA	*A. baumannii*	>1024/>1024	256/256	128/2	Y	N	N
		>1024/>1024	128/128	128/4	Y	N	N
		>1024/>1024	1024/1024	>1024/>1024	Y	N	N
		>1024/>1024	64/64	256/≤0.5	Y	Y	Y
		>1024/>1024	>1024/>1024	>1024/2	Y	N	N
		>1024/>1024	64/64	>1024/4	Y	N	N
		>1024/>1024	64/64	>1024/8	Y	N	N
		>1024/>1024	8/8	16/≤0.5	Y	N	N
		>1024/>1024	32/16	>1024/2	Y	N	N
		>1024/>1024	64/4	512/8	Y	N	N
		>1024/128	512/512	>1024/>1024	Y	N	N
		>1024/>1024	128/128	128/8	Y	N	N
		>1024/>1024	128/128	64/8	Y	N	N
	*C. freundii*	>1024/>1024	>1024/>1024	>1024/1024	Y	Y	Y
	*E. cloacae*	>1024/8	512/512	>1024/>1024	Y	Y	Y
		>1024/>1024	256/≤0.5	>1024/≤0.5	Y	Y	Y
		>1024/>1024	64/≤0.5	1024/≤0.5	Y	Y	Y
		>1024/>1024	>1024/1	>1024/256	Y	Y	Y
		>1024/>1024	64/≤0.5	>1024/≤0.5	Y	Y	Y
	*E. coli*	>1024/>1024	>1024/>1024	>1024/>1024	Y	Y	Y
		>1024/>1024	>1024/>1024	>1024/>1024	Y	Y	Y
		>1024/1024	16/≤0.5	≤0.5/≤0.5	Y	Y	Y
		>1024/>1024	512/512	>1024/≤0.5	Y	Y	Y
		>1024/>1024	256/256	>1024/>1024	Y	Y	Y
		>1024/>1024	1024/1024	>1024/>1024	Y	Y	Y
		>1024/>1024	1024/1024	>1024/>1024	Y	Y	Y
	*K. aerogenes*	>1024/≤0.5	32/32	1024/64	Y	Y	Y
	*K. ozaenae*	>1024/>1024	256/≤0.5	128/≤0.5	Y	Y	Y
	*K. pneumoniae*	>1024/>1024	64/64	>1024/≤0.5	Y	Y	Y
		>1024/>1024	>1024/>1024	>1024/>1024	Y	Y	Y
		>1024/512	512/512	>1024/>1024	Y	Y	Y
		>1024/>1024	64/32	>1024/1	Y	Y	Y
		>1024/128	4/4	>1024/256	Y	Y	Y
		>1024/>1024	>1024/>1024	>1024/>1024	N	N	N
		>1024/>1024	1024/1024	>1024/512	Y	Y	Y
		>1024/>1024	1024/16	>1024/1024	Y	Y	Y
		-	-	-		Y	Y
		>1024/>1024	256/256	>1024/>1024	Y	Y	Y
		>1024/>1024	64/64	>1024/1	Y	Y	Y
		>1024/>1024	128/128	>1024/>1024	Y	Y	Y
		>1024/128	256/256	>1024/512	Y	Y	Y
		>1024/>1024	16/8	>1024/8	Y	Y	Y
		>1024/>1024	1024/2	>1024/8	Y	Y	Y
		>1024/>1024	>1024/32	>1024/>1024	Y	Y	Y
		>1024/>1024	512/8	512/2	Y	Y	Y
		>1024/>1024	>1024/>1024	>1024/>1024	Y	Y	Y
		>1024/>1024	>1024/>1024	1024/1024		Y	Y
		>1024/>1024	512/16	>1024/>1024	Y	Y	Y
		>1024/>1024	1024/32	>1024/16	Y	Y	Y
		>1024/1024	8/2	>1024/16	Y	Y	Y
		>1024/≤0.5	512/512	>1024/256	Y	Y	Y
		>1024/>1024	1024/1024	>1024/>1024	Y	Y	Y
		>1024/>1024	512/512	>1024/>1024	Y	Y	Y
		>1024/>1024	1024/1024	>1024/>1024	Y	Y	Y
		>1024/>1024	>1024/>1024	>1024/>1024	Y	Y	Y
		>1024/>1024	512/512	>1024/512	Y	Y	Y
		>1024/>1024	1024/1024	>1024/>1024	Y	Y	Y
		>1024/>1024	64/32	≤0.5/≤0.5	Y	Y	Y
	*M. morganii*	>1024/>1024	16/8	>1024/>1024	Y	N	Y
	*P. aeruginosa*	>1024/>1024	>1024/>1024	>1024/>1024	Y	N	Y
		>1024/>1024	>1024/>1024	>1024/>1024	Y	N	Y
		>1024/>1024	256/16	>1024/1	Y	N	Y
		>1024/>1024	1024/1024	>1024/1024	Y	N	Y
		>1024/>1024	64/64	>1024/512	Y	N	Y
		>1024/>1024	64/64	16/4	Y	N	Y
		>1024/>1024	512/512	256/128	Y	N	Y
		>1024/>1024	>1024/>1024	>1024/>1024	Y	N	Y
		>1024/>1024	64/64	32/2	Y	N	Y
		>1024/>1024	>1024/1024	>1024/>1024	Y	N	Y
		>1024/>1024	512/512	>1024/64	Y	N	Y
		>1024/>1024	256/256	64/16	Y	N	Y
	*P. mirabilis*	512/64	4/4	128/128	Y	Y	Y

Carba, carbapenemase; β-lactams (BLs) tested include CA (clavulanate), VB (vaborbactam), AV (avibactam); Partner β-lactamase inhibitors (BLIs) tested include CA (clavulanate), VB (vaborbactam), AV (avibactam); −, Not available.

**Figure 1:**
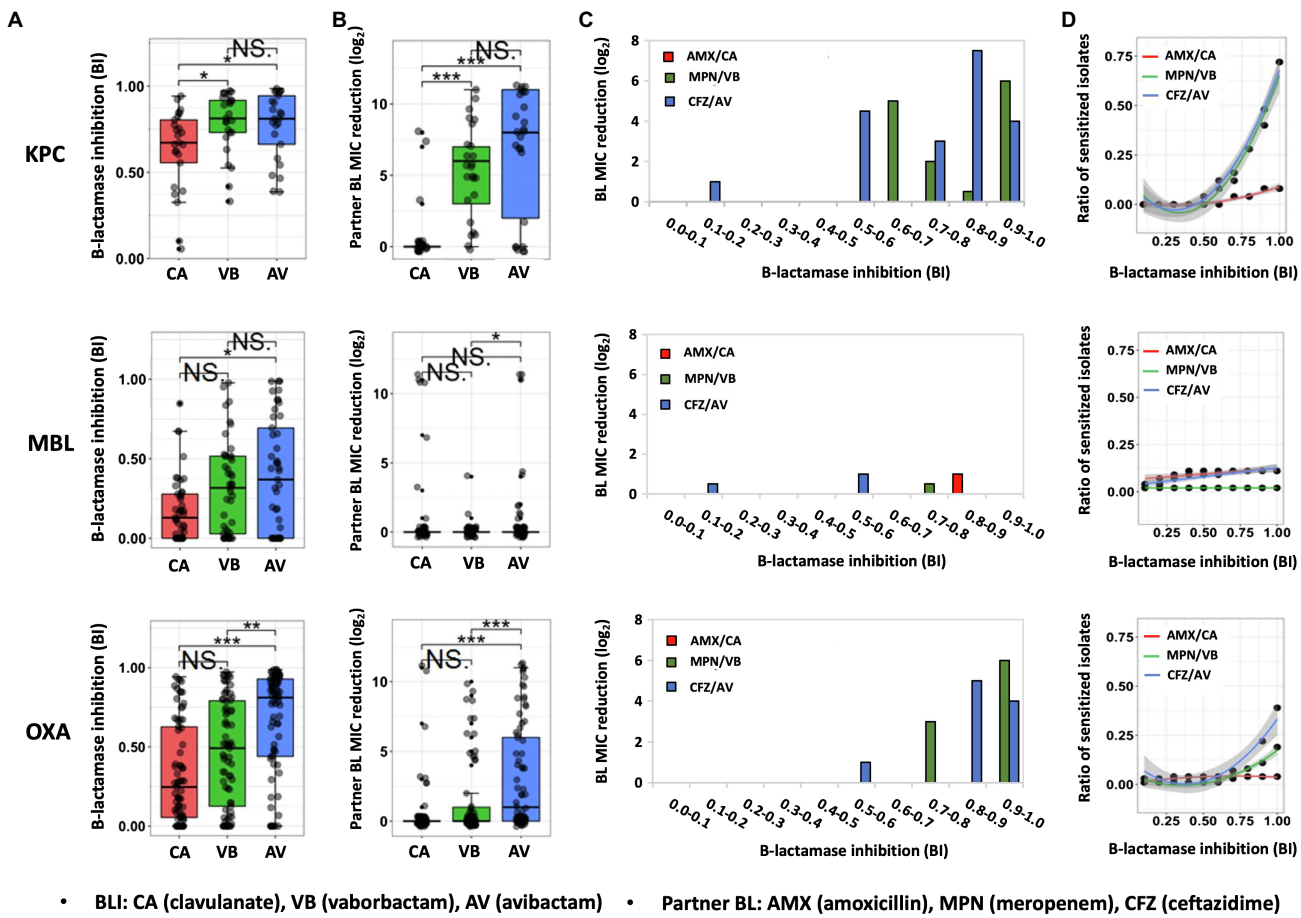
Quantitative relationship between BLI’s β-lactamase inhibition and its partner BL’s susceptibility restoration in CPO human isolates. Distribution of β-lactamase inhibition **(A)** and the resulted partner BL susceptibility restoration **(B)** of three clinically representative BLIs (50μM) in CPO human isolates containing KPC carbapenemase, MBL carbapenemase, OXA carbapenemase. Partner BL MIC reduction on average **(C)** and the ratio of sensitized isolates (BL MIC reduction ≥4 times) **(D)** by the function of β-lactamase inhibition among different molecular groups of CPO human isolates. BI, β-lactamase inhibition index; NS., no significant difference; ^*^, significant difference (*P* value of <0.05); ^**^, significant difference (*P* value of <0.01); ^***^, significant difference (*P* value of <0.001).

Upon the same BLI exposure, the β-lactamase inhibition, quantified by BI, varies widely from one isolate to another within the same carbapenemase molecular group of CPO (Figure 1A). Despite the individual heterogeneity, each carbapenemase molecular group of CPO has its own superior BLI (s): KPC isolates have high BIs induced by avibactam/vaborbactam and a lower BI led by clavulanate; MBL isolates are overall resistant to all tested BLIs (BIs <0.5); among OXA isolates, the most potent β-lactamase inhibition is induced by avibactam followed by vaborbactam and clavulanate.

Same to the β-lactamase inhibition, the BL susceptibility restoration in response to each BLI has also shown a great extent of individual heterogeneity in the same carbapenemase molecular group of CPO (Figure 1B). Significantly, a higher degree of β-lactamase inhibition (Figure 1A) is often corresponding to a bigger scale of partner BL MIC reduction (Figure 1B). For example, avibactam and vaborbactam, which lead to a stronger β-lactamase inhibition compared to clavulanate among KPC islates, result in a bigger decrease of partner BL MIC versus clavulanate among KPC isolates. As the most potent BLI for OXA type of CPO isolates, avibactam result in the highest partner BL MIC reduction in comparison with the other two tested BLIs among the OXA-producing CPO. These data indicate that BLI’s β-lactamase inhibitory efficacy is positively correlated with its partner BL’s susceptibility restoration. Such correlation is further supported by the increase of the BL susceptibility restoration (Figure 1C) and the ratio of the sensitized isolates by the function of BI (Figure 1D) for both KPC-produing and OXA-producing CPOs.

It is noteworthy that the increase of β-lactamase inhibition of a BLI is not proportional to its partner BL’s susceptibility restoration, as illustrated in Figures 1C,D. Significantly, when BI is low (<0.5), both the BL susceptibility restoration (Figure 1C) and the ratio of sensitized isolates (Figure 1D) are universally low for all BLI/CPO groups, suggesting that, without sufficient BLI dosing, there is no carbapenemase molecular identity-dependent superiority when choosing among BL/BLI agents. This is especially true for MBL isolates which none of the tested BLIs could effectively inhibit (median BIs, 0.1–0.4).

Intriguingly, even in isolates with a high degree of BLI-induced β-lactamase inhibition (BI>0.9), 50% (3/6) of CPO isolates had their MICs unchanged to amoxicillin, 50% (11/22) to ceftazidime and 35% (14/40) to meropenem. These isolates all carry additional BL resistance mechanisms besides β-lactamases, suggesting that BL resistance mechanisms independent of β-lactamase also play a significant role in the efficacy of BL/BLI to CPO. The non-proportional increase of the BL susceptibility by BLI’s β-lactamase inhibition shown in Figures 1C,D further supports this conclusion.

## Discussion

By quantifying BLI-induced β-lactamase inhibition in diverse CPO isolates, this study demonstrated the variation of BLI activity with carbapenemase molecular classes, supporting the carbapenemase identity derived BL/BLI treatment guidelines currently proposed for CPO (Pogue et al., 2019). On the other hand, our data revealed the substantial BLI response heterogeneity from isolates within the same carbapenemase molecular group. Important contributors to this heterogeneity might include variations in carbapenemase subtype/expression status and the co-existence of non-carbapenemase β-lactamases among CPO (Bush and Bradford, 2020). Therefore, the choice of BL/BLI for CPO infection should be personalized upon the entire β-lactamase background, rather than the carbapenemase identity alone, perhaps by further exploiting genetic sequencing information or utilizing other quantitative β-lactamase inhibition assays like FIBA.

This study provides for the first time, a quantitative insight into the correlation between β-lactamase inhibition and BL activity restoration against CPOs. The positive correlation illustrated here supports BL/BLI agents as effective CPO treatments, in line with the results of several clinical trials recently performed (Van Duin et al., 2018; Pogue et al., 2020). Beyond this, our data demonstrate that the anti-CPO success of a BL/BLI depends on the completeness of a BLI’s β-lactamase inhibition, motivating the need to alter BLI dosing to suit the specific β-lactamases in the clinical isolate. Therefore, BLI as an independent treatment adjuvant merits future consideration. To select the most suitable BLI, the structures and the resultant β-lactamase inhibitory mechanisms and profiles of the available BLIs have to be carefully compared in order to achieve the best clinical outcome. In addition, whether an BLI itself has antimicrobial activity besides β-lactamase inhibitory activity has also to be taken in account. To quantify the percentage of CPOs whose BL MICs are significantly changed by the introduction of BLIs, a CPO was considered sensitized by a BLI in this study if its BL MIC was reduced no less than 4 times after the BLI inclusion. However, it is of note that a reduction in the MIC by 4-fold or more may not be sufficient to change one CPO’s clinical susceptibility as bacteria are classified as susceptible (potentially treatable) or resistant (probably not treatable) to a particular agent based on whether the MIC of this agent falls below or above a clinical breakpoint.

Our results, in line with other findings (Sun et al., 2017; Cabot et al., 2018; Dulyayangkul et al., 2020; Sadek et al., 2020; Gomis-Font et al., 2021), suggested that substantial β-lactamase inhibition generated by the use of BLI may not significantly improve the susceptibility of CPO toward its partner BL due to the presence of BL resistance mechanisms irrelated to β-lactamase production. Thus, besides BLI inhibitor efficacy, BL/BLI choice should also be customized based on the other relevant BL resistance mechanisms of CPO. However, the current molecular tests for BL resistance are still mainly based on the detection of the bacterial β-lactamase production (Evans et al., 2019). Therefore, deciphering the β-lactamase-independent BL resistance mechanisms that significantly influence a BL/BLI’s efficacy to clinically significant pathogens, such as CPO, is urgently needed.

In summary, by quantitatively evaluating BLIs’ contribution to CPO treatments with BLs, this study recommends personalization of BL/BLI usage based on the whole resistance backgrounds of the specific CPO case. Specifically, BLI choice and dosing should be guided by quantitative tools that can evaluate the inhibition across the entire β-lactamase background of the CPO upon BLI treatment. Furthermore, rapid molecular diagnostics for BLI resistances, especially those sensitive to non-β-lactamase resistance mechanisms, should be exploited to prevent ineffective BL/BLI treatment. Though the scope of this study was limited to CPO, the insights acquired here are adaptable to all bacterial pathogens for which BL/BLI agents could be effective.

## Data Availability Statement

The original contributions presented in the study are included in the article/supplementary material, further inquiries can be directed to the corresponding author.

## Author Contributions

YF conceived of the presented idea and drafted the manuscript. YF and AV conducted the experiments. SK and MJ performed the statistical analysis. TH supervised the project. All authors discussed the results and contributed to the finalization of the manuscript.

## Funding

This study was supported by the Military Medicine Photonics Program from the US Department of Defense/Air Force (FA9550-16\1-0479), and the Netherlands Organization for Scientific Research (NWO) Rubicon program (452172009).

## Conflict of Interest

The authors declare that the research was conducted in the absence of any commercial or financial relationships that could be construed as a potential conflict of interest.

## Publisher’s Note

All claims expressed in this article are solely those of the authors and do not necessarily represent those of their affiliated organizations, or those of the publisher, the editors and the reviewers. Any product that may be evaluated in this article, or claim that may be made by its manufacturer, is not guaranteed or endorsed by the publisher.
